# Supplementary material for: Mild respiratory SARS-CoV-2 infection can cause multi-lineage cellular dysregulation and myelin loss in the brain
Source: bioRxiv. 2022 Jan 10:2022.01.07.475453. Preprint. [Version 1] doi: 10.1101/2022.01.07.475453 (PMC8764721; doi:10.1101/2022.01.07.475453)
Supplement: 1 [file NIHPP2022.01.07.475453V1-supplement-1.pdf]

bioRxiv preprint doi: https://doi.org/10.1101/2022.01.07.475453; this version posted January 10, 2022. The copyright holder for this preprint (which was not certified by peer review) is the author/funder, who has granted bioRxiv a license to display the preprint in perpetuity. It is made available under aCC-BY 4.0 International license.

Supplementary Figure 1. Neuroinvasive SARS-CoV-2 mouse model as a positive control

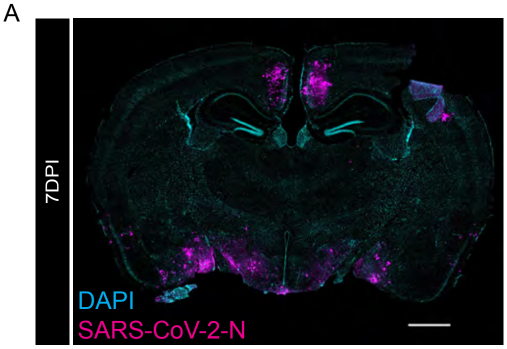

Supplementary Figure 1. Neuroinvasive SARS-CoV-2 mouse model as a positive control for nucleocapsid protein immunostaining

(A) Confocal micrograph of coronal section of mouse brain, illustrating SARS-CoV-2 nucleocapsid protein (SARS-CoV-2-N) 7-days post-infection (SARS-CoV-2-N, magenta; DAPI, cyan). Scale bar 1mm.

Related to Figure 1.

bioRxiv preprint doi: https://doi.org/10.1101/2022.01.07.475453; this version posted January 10, 2022. The copyright holder for this preprint (which was not certified by peer review) is the author/funder, who has granted bioRxiv a license to display the preprint in perpetuity. It is made available under aCC-BY 4.0 International license.

Supplementary Figure 2. Evidence of SARS-CoV-2 infection in lung

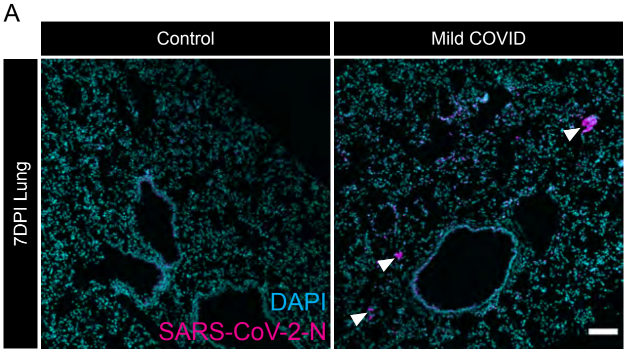

Supplementary Figure 2. Evidence of SARS-CoV-2 infection in lung of mild respiratory COVID mouse model

(A) Representative confocal micrographs of SARS-CoV-2 nucleocapsid protein (SARS-CoV-2-N, magenta; DAPI, cyan) in mouse lung 7-days post-infection. Arrowheads highlight SARS-CoV-2-N nucleocapsid protein immunostaining. Scale bar 100µm.
Related to Figure 1.

bioRxiv preprint doi: https://doi.org/10.1101/2022.01.07.475453; this version posted January 10, 2022. The copyright holder for this preprint (which was not certified by peer review) is the author/funder, who has granted bioRxiv a license to display the preprint in perpetuity. It is made available under aCC-BY 4.0 International license.

Supplementary Figure 3. White matter-selective microglial reactivity in humans with SARS-CoV-2 infection

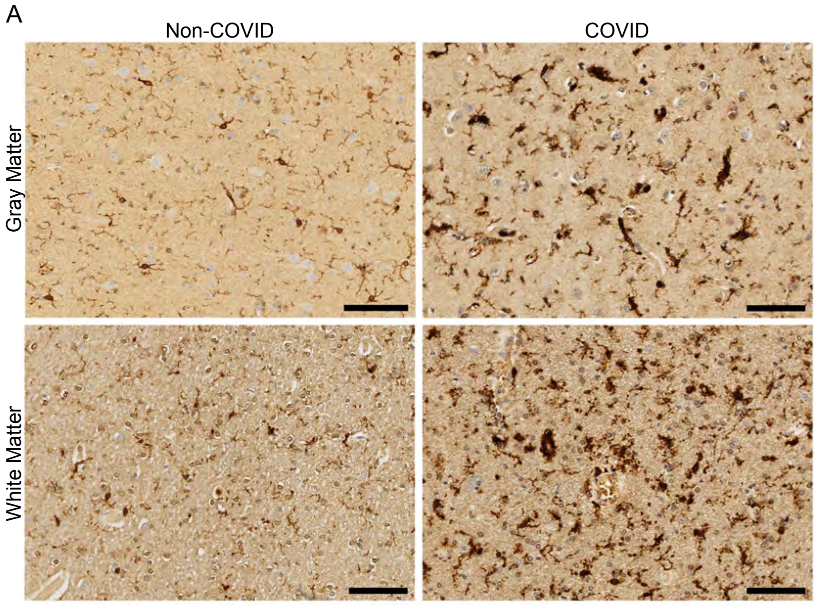

Supplementary Figure 3. White matter-selective microglial reactivity in humans with SARS-CoV-2 infection

(A) Representative micrographs of IBA1 immunostaining (brown) in the cerebral cortex (gray matter) or subcortical white matter of human subjects with or without COVID. Scale bars 100μm. Related to Figure 3.

bioRxiv preprint doi: https://doi.org/10.1101/2022.01.07.475453; this version posted January 10, 2022. The copyright holder for this preprint (which was not certified by peer review) is the author/funder, who has granted bioRxiv a license to display the preprint in perpetuity. It is made available under aCC-BY 4.0 International license.

Supplementary Figure 4. CCL11 levels after mild respiratory SARS-CoV-2 infection

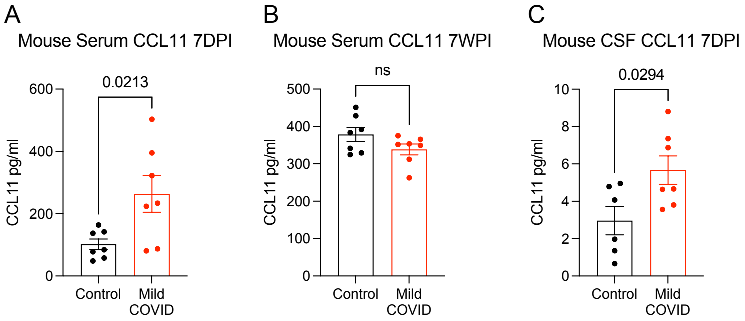

Supplementary Figure 4. CCL11 levels after mild respiratory SARS-CoV-2 infection
(A and B) Serum levels of CCL11 from CD1 mice 7-days post-infection (A) and 7-weeks post-infection (B). n=7 mice per group.
(C) CCL11 levels in CSF of CD1 mice 7-days post-infection. n=7 mice per group.
Data shown as mean +/- SEM; each dot represents an individual mouse; P values shown on figure panels; ns p>0.05; two-tailed unpaired t-test.
Data shown in heatmap form in Figure 1. Related to Figures 1 and 4.

bioRxiv preprint doi: https://doi.org/10.1101/2022.01.07.475453; this version posted January 10, 2022. The copyright holder for this preprint (which was not certified by peer review) is the author/funder, who has granted bioRxiv a license to display the preprint in perpetuity. It is made available under aCC-BY 4.0 International license.

Supplementary Figure 5. Validation of oligodendrocyte loss after SARS-CoV-2 infection

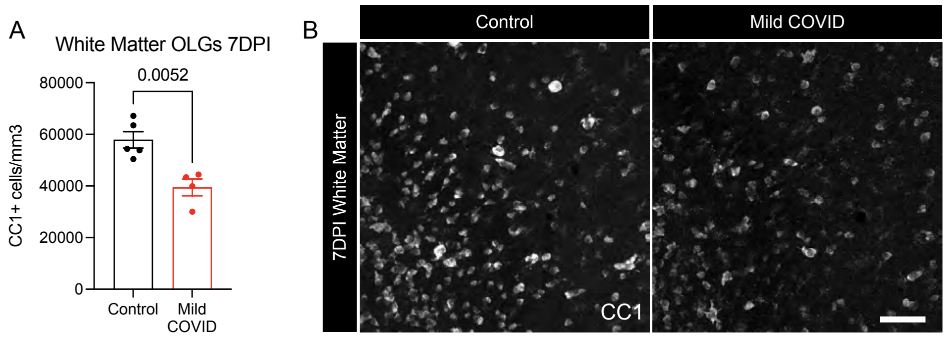

Supplementary Figure 5. Validation of oligodendrocyte loss after mild respiratory SARS-CoV-2 infection

(A) Oligodendrocyte (CC1+) quantification in the cingulum of the corpus callosum of CD1 mice 7-days post-infection.

(B) Representative confocal micrographs of oligodendrocytes (CC1, white) in the cingulum of the corpus callosum of CD1 mice 7-days post-infection. Scale bar 50µm.

Data shown as mean +/- SEM; each dot represents an individual mouse; unpaired, two-tailed t-test. P value shown on figure panel.

Related to Figure 5.

bioRxiv preprint doi: https://doi.org/10.1101/2022.01.07.475453; this version posted January 10, 2022. The copyright holder for this preprint (which was not certified by peer review) is the author/funder, who has granted bioRxiv a license to display the preprint in perpetuity. It is made available under aCC-BY 4.0 International license.

Supplementary Figure 6. Myelin ultrastructure after respiratory SARS-CoV-2 infection

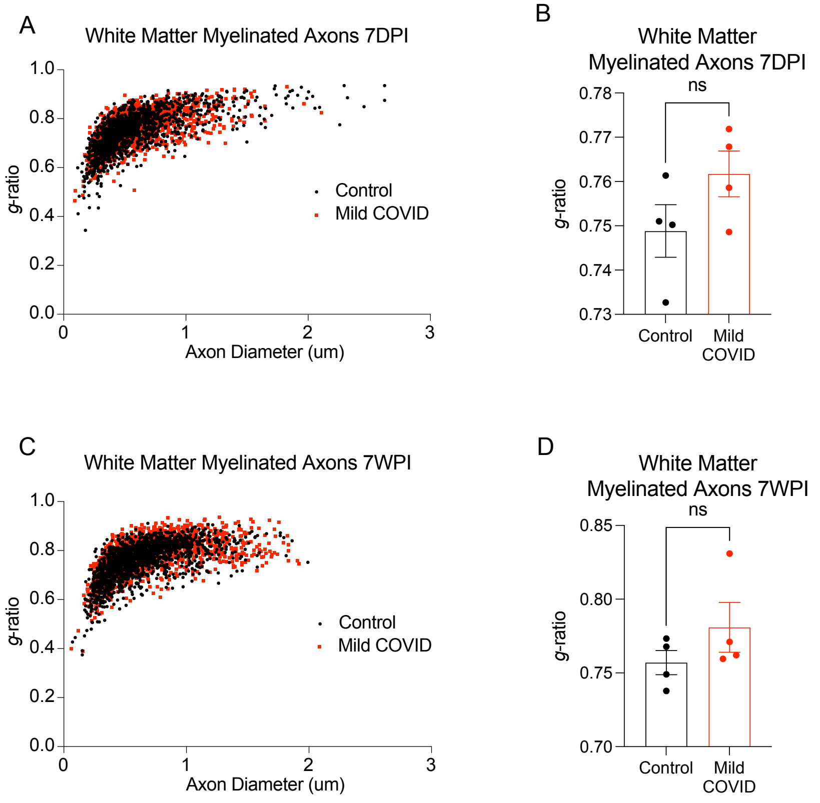

Supplementary Figure 6. Myelin sheath thickness after mild respiratory SARS-CoV-2 infection

(A) Scatter plots of *g*-ratio relative to axon diameter 7-days-post infection. Black dots, control axons; red dots, axons from mice with mild respiratory COVID.

(B) Cumulative *g*-ratios of myelinated axons per animal at 7-days-post infection. Each dot represents an individual mouse.

(C) Scatter plots of *g*-ratio relative to axon diameter 7-weeks-post infection. Black dots, control axons; red dots, axons from mice with mild respiratory COVID.

(D) Cumulative *g*-ratios of myelinated axons per animal at 7-weeks-post infection. Each dot represents an individual mouse.

Data in B and D shown as mean +/- SEM; n=4 mice per group; ns $p>0.05$ by two-tailed, unpaired t-test.

Related to Figure 5.

bioRxiv preprint doi: https://doi.org/10.1101/2022.01.07.475453; this version posted January 10, 2022. The copyright holder for this preprint (which was not certified by peer review) is the author/funder, who has granted bioRxiv a license to display the preprint in perpetuity. It is made available under aCC-BY 4.0 International license.

Supplementary Table 1. COVID-19 subject and non-COVID-19 control subject characteristics

Case	Age/Sex	Past Medical Hx	Recent Hx	Days from symptom onset to death	Autopsy findings	Post-mortem Interval (hours)
COVID Case 1	73/M	HTN, obesity	Pneumonia, renal failure, pericardial effusion, arrythmia, hospitalized for 8 days intubated for 3 days	16 days	Gross Autopsy Findings: Severe pulmonary congestion with multifocal hemorrhages, pleural effusion, renal cortical pallor, fibrosis and calcification on epicardial surface<br>Brain Autopsy Findings: Perivascular infiltrates, Eosinophilic degenerating neurons, Perivascular pallor in the cerebellar white matter	38
COVID Case 2	39/M	Methamphetamine use disorder, cardiac and kidney disease	Cardiac, renal and respiratory failure, Pulmonary emboli, hospitalized for 3 days	2 days	Gross Autopsy Findings: Cardiac hypertrophy, pulmonary and renal infarcts<br>Brain Autopsy Findings: Perivascular infiltrates	36
COVID Case 3	50/M	DM, sciatica, back pain	Cough 3-5 days, found dead at home, PM swab +	days	Gross Autopsy Findings: Acute DAD, HASCVD, fatty liver, nephrosclerosis<br>Brain Autopsy Findings: Scattered acutely ischemic neurons	33
COVID Case 4	39/M	Drug use disorder	Unknown, found dead in subway, PM swab +	hours to days (presumed)	Gross Autopsy Findings: Acute lung injury, toxicology + fentanyl, heroin, alcohol, fatty liver, LVH<br>Brain Autopsy Findings: Acute neuronal ischemia, focal gliosis of hippocampus	34
COVID Case 5	58/F	DM, Obesity, asthma, schizophrenia, had hospitalization with intubation for 1 month, discharged 1 month earlier	Unknown, found dead at home, PM swab +	days to weeks (presumed)	Gross Autopsy Findings: Acute lung injury, fatty liver, diabetic kidney disease<br>Brain Autopsy Findings: Microglial prominence and focal encephalitis (neuronophagia) of insula, basal nuclei, pons, medulla; Incidental capillary telangiectasia in pons and basal ganglia	29
COVID Case 6	58/F	Obesity	GI symptoms few days, found dead at home, PM swab +	days	Gross Autopsy Findings: Acute DAD and fibrin thrombi, LVH, nephrosclerosis<br>Brain Autopsy Findings: Focal mineralization of thalamic neurons	30
COVID Case 7	24/M	Flu-like symptoms 3 weeks prior	Sudden death, PM swab +	3 weeks	Gross Autopsy Findings: Acute lung injury<br>Brain Autopsy Findings: Sparse perivascular parenchymal and leptomeningeal lymphocytes and microglial nodules in medulla	13
COVID Case 8	55/M	HTN, DM, HLD, obesity, depression	Flu-like symptoms for few days, agitated delirium, subdued by force, hospitalized 6 days, hospital swab and PM swab +	days to weeks	Gross Autopsy Findings: Acute DAD with fibrin thrombi, fatty liver disease and fibrosis, gun shot wounds to limbs<br>Brain Autopsy Findings: Acute neuronal ischemia and microglial reaction, hippocampus, and temporal neocortex	105
COVID Case 9	54/M	Old TBI from assault 3 years prior, post-traumatic seizures, DM, in nursing home	Recent flu-like symptoms, nursing home swab +	Days	Gross Autopsy Findings: Aspiration pneumonia<br>Brain Autopsy Findings: Subdural neomembranes, old contusions, slight perivascular lymphocytes in pons	50
Control Case 1	54/M	HTN, CAD with prior MI and coronary stent placement, smoking (1ppd for ~30 yrs, quit 5 years prior to death), colorectal tumor resection (benign)	N/A	N/A	Brain Autopsy Findings: No significant brain pathology	15
Control Case 2	54/M	HTN, HLD, CAD with previous MI s/p pacemaker placement and CABG, CHF, atrial fibrillation Alcohol	N/A	N/A	Brain Autopsy Findings: Hypoxic-ischemic encephalopathy, cerebrovascular disease (arteriolosclerosis)	48

bioRxiv preprint doi: https://doi.org/10.1101/2022.01.07.475453; this version posted January 10, 2022. The copyright holder for this preprint (which was not certified by peer review) is the author/funder, who has granted bioRxiv a license to display the preprint in perpetuity. It is made available under aCC-BY 4.0 International license.

		abuse, cirrhosis, end-stage kidney disease requiring dialysis, severe peripheral vascular disease complicated by lower limb gangrene requiring left lower extremity amputation				
Control Case 3	43/M	Bipolar disorder, schizophrenia, depression, PTSD, seasonal affective disorder, sickle cell trait, HLD, smoking (1ppd for ~25 years)	N/A	N/A	Brain Autopsy Findings: No significant brain pathology	28
Control Case 4	70/M	Headaches, alcohol abuse, smoking (reported "5ppd since teens")	N/A	N/A	Brain Autopsy Findings: Cerebrovascular disease (arteriolosclerosis), age-related tauopathy	N/A
Control Case 5	65/M	HTN, asthma, insulin-dependent diabetes, cocaine use, 1/2ppd smoker, unexplained mental decline and gait instability	N/A	N/A	Brain Autopsy Findings: Cerebrovascular disease (atherosclerosis, arteriolosclerosis), Lewy body disease/Parkinson's	38

Legend: N/A, not available/applicable. Abbreviations: PMI, postmortem interval; CMV, cytomegalovirus; COPD, chronic obstructive pulmonary disease; DAD, diffuse alveolar damage, HTN, hypertension; CAD, coronary artery disease; MI, myocardial infarction; HLD, hyperlipidemia; CABG, coronary artery bypass grafting; CHF, congestive heart failure; PTSD, post-traumatic stress disorder.
Related to Figure 3.
